# Verbal Cuing Is Not the Path to Enlightenment. Psychological Effects of a 10-Session Hatha Yoga Practice

**DOI:** 10.3389/fpsyg.2020.01375

**Published:** 2020-07-02

**Authors:** Barbara Csala, Eszter Ferentzi, Benedek T. Tihanyi, Raechel Drew, Ferenc Köteles

**Affiliations:** ^1^Doctoral School of Psychology, ELTE Eötvös Loránd University, Budapest, Hungary; ^2^Institute of Health Promotion and Sport Sciences, ELTE Eötvös Loránd University, Budapest, Hungary

**Keywords:** yoga, verbal instruction, spirituality, mindfulness, affect, body awareness

## Abstract

Verbal instructions provided during yoga classes can differ substantially. Yoga instructors might choose to focus on the physical aspects of yoga (e.g., by emphasizing the characteristics of the poses), or they might take a more spiritual approach (e.g., by mentioning energy flow and chakras). The present study investigated the effects of verbal cues during yoga practice on various psychological measures. Eighty-four female students (22.0 ± 3.80 years) participated in the study. Two groups attended a beginner level hatha yoga course in which physically identical exercise was accompanied by different verbal cues. The so-called “Sport group” (*N* = 27) received instructions referring primarily to the physical aspects of yoga practice, while the “Spiritual group” (*N* = 23) was additionally provided with philosophical and spiritual information. A control group (*N* = 34) did not receive any intervention. Mindfulness, body awareness, spirituality, and affect were assessed 1 week before and after the training. 2 × 3 mixed (time × intervention) ANOVAs did not show an interaction effect for any of the variables. However, when the two yoga groups were merged and compared to the control group, we found that spirituality increased, and negative affect decreased among yoga participants. In conclusion, yoga practice might influence psychological functioning through its physical components, independent of the style of verbal instructions provided.

## Introduction

Traditionally, yoga has comprised various physical, mental, moral, and spiritual practices aimed at improving holistic health, well-being, and self-awareness (Iyengar, 1991; Impett et al., 2006; Ross and Thomas, 2010). In Western society, however, it is often interpreted purely as a form of physical fitness (MacDonald, 2013; Sarbacker, 2014). Yoga classes have recently increased in popularity, and participants exhibit a number of health benefits when participating in regular practice (e.g., Rocha et al., 2012; Groessl et al., 2015; Riley and Park, 2015; Dwivedi and Tyagi, 2016), however, classes show diversity in style and composition (Ross and Thomas, 2010; Park et al., 2018). As classes may include detailed verbal information on physical (e.g., precise description of the body posture), psychological (e.g., awareness of different bodily sensations and occurring thoughts), and spiritual (e.g., focusing on pure consciousness or oneness during postures) aspects of yoga, the verbal cues provided by a yoga instructor can facilitate a variety of physical and mental experiences during practice (Emerson et al., 2009).

Hatha yoga is a prominent style of yoga practice in Western society. It emphasizes physical postures (asana), breathing exercises (pranayama), and relaxation and meditation techniques (Devereux, 1994). Hatha yoga differs from other forms of physical activity in several ways: (1) its specific body postures including relaxation poses, (2) breath regulation, (3) sustained maintenance of the postures, and (4) the requirement of constant non-judgmental attention during practice (Veda Bharati, 1985; Yesudian and Haich, 1992; Impett et al., 2006; Govindaraj et al., 2016). The latter three characteristics are shared by other so-called mind–body exercises (Mehling et al., 2011; Patwardhan, 2017). As hatha yoga is typically used in beginner yoga classes, we have chosen to focus on this style of yoga practice in the present research. We propose that qualitative differences in verbal instructions during practice may explain the variance in the effect of hatha yoga practice on psychological constructs that are likely to be affected by participation in yoga classes, such as spirituality, body awareness, mindfulness, and affect (Delaney and Anthis, 2010; Büssing et al., 2012a; Csala et al., 2017; Hendriks et al., 2017; Domingues, 2018).

The fundamental importance of spirituality in yoga is widely accepted and emphasized (Iyengar, 1991; Baktay, 1992; Büssing et al., 2012a). Spirituality has been defined as a universal, innate, human-specific phenomenon – a belief in and search for something sacred beyond the material world (Piedmont and Leach, 2002; Hill and Pargament, 2003; Emmons, 2006; Tomcsányi et al., 2011). Although extensive literature indicates that spirituality is highly relevant to human functioning and health (Bergin, 1997; MacDonald, 2013), only a few empirical studies have investigated the contribution of spirituality to the positive effects of yoga practice to date (Büssing et al., 2012a; MacDonald, 2013). In traditional yogic terminology, spirituality refers to the spirit (i.e., the most inner core of humans), which can also be interpreted as pure consciousness (Rama et al., 1976; Baktay, 1992; Swartz, 2015). The final aim of spiritual practices in yoga is to “be absorbed by the spirit within,” which corresponds to the universal spirit (i.e., universal consciousness). This experience is often referred to as “oneness,” “union,” or “highest state of consciousness” (Rama et al., 1976; Iyengar, 1991; Swartz, 2015). From a scientific point of view, however, spirituality refers to a broad construct with facets, such as cognitive orientation toward spirituality, experiential/phenomenological dimension, existential well-being, paranormal beliefs, or religiousness. No widely accepted conceptualization and measurement of spirituality exists; nevertheless, a wide range of instruments seem to be promising for yoga research (MacDonald and Friedman, 2009; MacDonald, 2013).

A recent cross-sectional investigation (Csala et al., 2017) reported that weekly frequency of yoga practice is associated with spiritual connection, even after controlling for gender, age, and education. An earlier study (Dittmann and Freedman, 2009) found that spiritual readiness (i.e., subjective relevance of ritual elements, and spiritual seeking and purpose) significantly correlated with higher level of body awareness, body responsiveness, and body satisfaction among women who practiced postural yoga regularly. However, it was unclear whether enhanced spirituality was the result of physical yoga practice, additional activities such as meditation, or other effects. An intervention study (Smith et al., 2011) investigated students with mild to moderate depression, anxiety, or stress. Participants were divided into three groups: yoga as exercise, integrated yoga (which also included yogic ethical guidelines), and a control group. Yoga intervention groups received a 7-week-long training with 14 classes (1 h twice a week), however, neither group showed significant changes in spiritual well-being. Authors proposed the inclusion of other measures of spirituality to explore whether yoga practice impacts this construct. In contrast, Büssing et al. (2012a) reported significant improvements in some aspects of spirituality (e.g., conscious interactions/compassion, religious orientation) among yoga teacher training participants after 6 months of intensive and complex yoga practice (including postures, breathing, relaxation, meditation, mantras, and philosophy lectures). Additionally, the authors noted that the constructs of spirituality, mindfulness, and mood appear to be strongly correlated.

Positive and negative affects (Watson et al., 1988) are two relatively independent dimensions of subjective well-being (Gyollai et al., 2011). Positive affect reflects a person’s level of joyfulness, activeness, and alertness, while negative affect denotes the level of subjective distress and unpleasurable feelings (Watson et al., 1988; Gyollai et al., 2011). Studies investigating the relationship between yoga practice and affect generally report favorable outcomes (West et al., 2004; Vadiraja et al., 2009; Meissner et al., 2016; Tihanyi et al., 2016b; Csala et al., 2017). For example, Meissner et al. (2016) investigated healthy female yoga practitioners and found a significant increase in positive affect and a decrease in negative affect after a brief intense yoga intervention when compared to those who maintained their original practice intensity. An intensive yoga training among early breast cancer patients resulted in similar beneficial results when compared to a supportive counseling group (Vadiraja et al., 2009). Conversely, a systematic review on yoga as a tool for mental health promotion (Domingues, 2018) concluded that results concerning positive and negative affect are equivocal.

The same review (Domingues, 2018) denoted that in four out of five studies, yoga interventions resulted in higher levels of mindfulness, which is the ability to be attentive to and aware of the present moment in a non-judgmental way (Brown and Ryan, 2003). An 8-week intervention for women with fibromyalgia (Curtis et al., 2011) reported significantly higher levels of mindfulness at the end of the program. These findings are supported by a number of cross-sectional studies (Brisbon and Lowery, 2011; Tihanyi et al., 2016a). Conversely, another systematic review (Hendriks et al., 2017) suggests that the positive effect of yoga practice on mindfulness is yet to be proven.

Similarly to mindfulness, body awareness (i.e., attentiveness to normal bodily processes) is positively associated with the weekly frequency of yoga practice and mediates the connection between yoga practice and well-being (Tihanyi et al., 2016a). In fact, body awareness can be considered a subcategory of mindfulness (Mehling et al., 2009, 2011; Hölzel et al., 2011). A recent study (Tihanyi et al., 2016b) examining advanced practitioners of different kinds of physical activity showed that yoga students have a significantly higher level of body awareness when compared to kung-fu, ballroom dance, and aerobics practitioners. Similar findings were reported in other studies as well (Daubenmier, 2005; Impett et al., 2006). One study (Delaney and Anthis, 2010) investigated the effects of different types of yoga classes concerning the extent to which they emphasized the “mind” aspects of yoga (e.g., meditation, mindfulness, chanting, yogic principles, and the non-physical self) and its “body” components (e.g., posture and fitness). Results showed that participants in classes with more emphasis on the mind reported higher levels of body awareness and satisfaction than those in classes with greater body focus.

The goal of the present study was to investigate the effect of verbal instructions during a brief beginner level traditional hatha yoga training on spirituality, body awareness, mindfulness, and affect among healthy female university participants. It was expected that yoga practice leads to favorable changes in the aforementioned constructs; moreover, participants receiving more holistic or spiritual verbal cuing would show greater improvements than those receiving instructions focused primarily on the physical aspects of the yoga practice. An additional aim of the study was to explore the potential intercorrelations among the changes in outcome variables.

## Materials and Methods

### Participants

*A priori* calculation (Faul et al., 2007) for a mixed 3 × 2 ANOVA with within-between interaction and a small effect size (*α* = 0.05, β = 0.8, *f* = 0.2) indicated a total sample size of 66 (i.e., *n* = 22 in each group). Overall, 115 participants were enrolled in the study. To avoid any potential gender effects, only female participants were eligible. Due to dropout or missing more than two yoga classes, the final sample included 84 female university students (mean age: 22.0 ± 3.80 years) with no previous experience in yoga. All participants were Hungarian university students, primarily enrolled in human and natural sciences. No exchange students were enrolled. Participants were divided into three groups: two yoga and one control group. The so-called “Sport group” (see the “Procedure” section for more details) consisted of 27 participants (mean age: 21.48 ± 2.08 years); the size of the “Spiritual group” was *n* = 23 (mean age: 21.43 ± 2.56 years). The control group encompassed 34 individuals (mean age: 22.79 ± 5.24 years).

Participants signed an informed consent form prior to participation. The research was permitted by the Research Ethics Committee of the Faculty of Education and Psychology at ELTE University.

### Procedure

Beginner level hatha yoga courses for female students were advertised on the university’s online registration platform (max. 14 people per group) at the beginning of three semesters. A course for control participants was advertised the same way. Applicants who chose to sign up for a yoga course were not aware that two yoga classes with different verbal instructions would be held. Thus, students who signed up for a yoga course were randomly assigned to one of the two intervention groups. Inclusion criteria were as follows for each group: (1) no previous experience with yoga; (2) intention to maintain current regular physical activity level during the study; (3) not starting new body focus related activities beyond the current study (such as relaxation or meditation); (4) avoiding any guided yoga techniques (e.g., recordings or videos) at home to control for an overall amount of practice; and (5) no known psychiatric diagnosis. After registering for the course, subjects were informed via e-mail about the conditions of participation. Questionnaires were completed online 1 week before and after the intervention.

The yoga course consisted of 10 weekly sessions, 1.5 h each, preceded and followed by physical, behavioral, and psychological measurements (the current paper reports only the psychological outcomes). Control students took part in the baseline and post-intervention measurements, but they did not receive any training or alternative intervention. Yoga classes were led by a certified (RYT500) female yoga instructor (BC, one of the authors) in Hungarian. Ethnicity and native language of the students and the teacher were the same. Each session focused on asana practice (all types of body postures such as sitting, kneeling, standing, balancing poses, forward and backward bends, supine and prone positions, inversions) and included a short body and breath focus at the beginning, as well as a short relaxation component (∼8–10 min in Savasana, the lying corpse position) at the end. Class composition was based on two highly regarded yoga books (Iyengar, 1991; Satyananda Saraswati, 2002; see Table 5 for class plans). Physical components of the classes (i.e., exact movements, order of postures) were identical for the two yoga groups, and the amount of verbal information provided during the sessions was equivalent. Scripts for the sessions were prepared before the start of the 10-week intervention and strictly followed by the yoga instructor throughout all three semesters. There were identical contents in the scripts for the two groups, however, there were also substantial differences in the instructions provided during sessions. Whereas the Sport group received instructions emphasizing the physical aspects of yoga practice (e.g., correct alignment of the body, which muscle is strengthened or stretched in the particular asana), the Spiritual group was provided with more holistic, i.e., philosophical and spiritual, descriptions and explanations (e.g., what is the energetic body, which chakra^1^ is activated by a specific posture and its corresponding mental, emotional characteristics, what is the final aim of yoga practice, “om” mantra chanting at the end of the class). For instance, Pawanmuktasana series (warming up exercises) were introduced in the following ways – Sport group: “The aim of these practices is to move the joints to open up them and gain better flexibility.” vs. Spiritual group: “The aim of these practices is to enhance flexibility and release energetical blockages from the body.” Similarly, in Trikonasana (standing hip opening posture), the instruction was as follows – Sport group: “Try to stretch your body in all directions, feel the opening of your hip. Feel the work of your side muscles and abdominal muscles.” vs. Spiritual group: “Stretch from your navel into all directions. Observe Manipura chakra, and quality of fire element, strength, endurance and will-power.” While holding the pose of Supta Pawanmuktasana (lying on the back with pulled up knees), the instruction was as follows – Sport group: “Most of the poses have an active effect on the spine, they strengthen or stretch it, like this one. The health of our spine is the foundation for healthy functioning: it provides the correct posture for the body which influences the proper operation of the inner organs. Aside from this, if the spine is healthy, it also keeps the nerves safe and sound.” vs. Spiritual group: “According to the ancient scripts, the aim of asana practice is to prepare the body for meditation. To be strong and flexible enough to hold a stable, comfortable sitting posture with a straight spine for a long time. Aside from this, it prepares the mind to be able to meditate in this pose, to focus and deepen within, and realize who we are in reality.” Components of the verbal instructions were evaluated using a recently developed questionnaire called the Essential Properties of Yoga Questionnaire (EPYQ) (Park et al., 2018). This assessment indicated that sessions for both yoga groups included the following components: *Breathwork; Physicality; Postures, Active; Postures, Restorative; Body Awareness; Individual Attention; Social Aspects; Meditation and Mindfulness;* and *Acceptance/Compassion* components. However, only one item on the *Acceptance/Compassion* subscale was included in the Sport group sessions, namely “Acceptance of your body while doing yoga.” This item pertains to avoiding overexertion in order to prevent injury. Other items on this subscale (e.g., “General thoughts of gratitude, love, kindness” and “Self-compassion”) were not featured in the Sport group but were incorporated in the Spiritual group sessions. Furthermore, the scripts for both groups mentioned *Health Benefits*, but in the Sport group, it referred to physical health, while in the Spiritual group, emotional, mental, and spiritual aspects of health were emphasized. Instructions for the Sport group acknowledged *Release* of the physical tension, while verbal cues for the Spiritual group also referred to *Mental and Emotional Awareness/Release, Spirituality*, and *Yoga Philosophy*. Neither set of instructions made reference to *Body Locks* (specific muscular contractions to induce energetic effects). To summarize, the similarities between the two intervention groups were the fundamental elements of traditional hatha yoga practice, including physicality and some psychological facets (e.g., focusing one’s awareness on breath, different physical sensations, and relaxation of the body). The main difference between the two groups was that the Sport group remained focused on these physical aspects of practice, while the Spiritual group received additional information about the philosophical and spiritual facets of yoga practice. Furthermore, social components, like small interactions between students, and individual attention (i.e., physical assessment of a student in posture) were also part of the experience in both groups. These inevitable characteristics of a guided physical group activity were equalized by the instructor as much as possible.

### Measurements

Demographic data included questions about age, education level, average physical activity level in the previous 3 months (measured by a five-point Likert scale), and a yes-or-no question concerning previous spiritual experiences (e.g., meditation, relaxation, religious practices, tai chi, reiki).

The *Body Awareness Questionnaire* (BAQ) (Shields et al., 1989) assesses the beliefs about one’s sensitivity to normal, non-emotive bodily processes and the ability to anticipate bodily reactions. It has 18 items measuring on a seven-point Likert scale (from “not at all true of me” to “very true of me”). Higher scores indicate a higher level of attentiveness to normal bodily processes. The scale is characterized by a good convergent and discriminant validity (Shields et al., 1989; Mehling et al., 2009). The Hungarian version of the scale also showed good validity and reliability (Köteles, 2014; Emanuelsen et al., 2015). In the present study, the Cronbach’s alpha value was 0.841 at the first and 0.865 at the second measurement.

The *Mindful Attention and Awareness Scale* (MAAS) (Brown and Ryan, 2003) measures the ability to focus on the present moment with an open, non-judgmental attitude. It consists of 15 inversely stated items using a six-point Likert scale (from “almost always” to “almost never”) assessing the automatic, inattentive actions of the respondent. Higher scores represent a higher level of mindfulness. The scale showed a negative correlation with reflective self-consciousness [assessed by the Self-Consciousness Scale (Fenigstein et al., 1975)], and only small to moderate relations to other dispositional self-awareness instruments, which means that MAAS measures a unique aspect of human consciousness (Brown and Ryan, 2003). The Hungarian version is in concordance with the original measure; it shows a good validity and has good internal consistency (Simor et al., 2013). In the present study, Cronbach’s alpha values were 0.859 and 0.850.

The *Positive and Negative Affect Schedule* (PANAS) (Watson et al., 1988) consists of two independent scales measured by 10–10 items on a five-point Likert scale (from “very slightly or not at all” to “extremely”). The Positive Affect scale indicates the extent to which a person feels active, alert, and enthusiastic. The Negative Affect scale measures subjective distress and unpleasant mood states like guilt or fear. Higher scores show higher positive or negative affect. The two scales are internally consistent and dispose of ideal convergent and discriminant validity since they showed expected correlations with expansive measures of underlying mood components. The Hungarian version of this scale had acceptable internal consistency (Gyollai et al., 2011). In the present study, Cronbach’s alpha coefficients were 0.843 and 0.882 for Positive Affect and 0.849 and 0.891 for Negative Affect, respectively.

The short version of the *Spiritual Connection Questionnaire* (SCQ-14) (Wheeler and Hyland, 2008) aims to assess spiritual connection as a subjective experience in a religion-independent way. It is a unidimensional measure capturing the feeling of connection with the universe and with others, and the happiness arising from the sense of connection. The 14 items are measured on a seven-point Likert scale (from “definitely not agree” to “definitely agree”); higher scores represent a higher level of spiritual connection. It shows negative correlation with self-enhancing values, such as power, security, and hedonism, and a positive correlation with self-transcendence values, like universalism (Wheeler and Hyland, 2008). The scale is freely accessible and usable. The Hungarian version showed good validity and high reliability (Csala and Köteles, unpublished manuscript); Cronbach’s alpha values were 0.930 and 0.943 in the present investigation.

### Data Analysis

Statistical analysis was conducted using the JASP V0.9.0.1. software (JASP Team, 2019). Homogeneity of groups with respect to baseline measurements was examined with one-way analysis of variance (ANOVA) and chi-square test. The effect of the intervention on the investigated variables was estimated using 2 × 3 mixed ANOVA (time x intervention). Since no differences between the two yoga groups were revealed, in the next step, the two yoga groups were merged and compared to the control group using 2 × 2 mixed ANOVAs. *Post hoc* analysis was conducted with Bonferroni correction (*p* < 0.05) in all cases. Changes with respect to the assessed variables in the merged yoga group were calculated by subtracting the baseline value from the respective post-intervention value for each participant. Associations between changes were estimated using Kendall’s tau-b coefficients. For all cases, Bayesian counterparts were also used. In the Bayesian analysis, probability of an alternative hypothesis compared to the null hypothesis is calculated; this is called Bayes Factor (BF_10_). A BF_10_ smaller than 0.33 indicates that the null hypothesis is more probable than the alternative hypothesis. BF_10_ between 1 and 3 yields weak or anecdotal support for the alternative hypothesis, whereas a value above 10 is considered strong support (Jarosz and Wiley, 2014).

The use of Bayesian statistics in behavioral science is strongly recommended by many authors, as it is able to overcome the often-mentioned limitations of the frequentist approach (e.g., Type 1 and 2 errors) and can give a more direct answer to research questions than the classic (aka frequentist) analysis (Dienes, 2011; Kline, 2013; Jarosz and Wiley, 2014).

## Results

### Demographic Data

There were no significant differences among the three groups with respect to age [*F*(2, 81) = 1.254; *p* = 0.291], education level [*F*(2, 81) = 1.734; *p* = 0.183], average physical activity level in the previous 3 months [*F*(2, 81) = 1.205; *p* = 0.305], and previous spiritual experiences [χ^2^(2) = 0.620; *p* = 0.733].

### Descriptive Statistics

Descriptive data of the measured psychological variables among the three groups and the merged yoga group, as well as the baseline comparison of the groups, are presented in Table 1. There were no significant differences between the three groups for any variable at baseline. Similarly, no significant differences emerged when the merged yoga group was compared to the control group.

**TABLE 1:** Descriptive data of the assessed variables before and after the intervention, and baseline comparison of the groups.

		Baseline			Baseline ANOVA			Post-intervention	
	Group	*N*	*M*	*SD*	*F*(*df*)	*p*	η *_*p*_*^2^	*M*	*SD*
MAAS	Sport	27	55.148	11.763	0.855 (81, 2)	0.429	0.021	56.741	12.171
	Spiritual	23	52.13	12.704				55.13	13.569
	Control	34	51.5	9.696				51.853	8.553
	Merged yoga	50	53.76	12.173	0.818 (82, 1)	0.368	0.010	56	12.725
BAQ	Sport	27	82.741	17.948	1.095 (81, 2)	0.339	0.026	83.333	14.631
	Spiritual	23	76.391	13.395				77.913	17.596
	Control	34	80.088	13.761				79.382	13.97
	Merged yoga	50	79.82	16.177	0.006 (82, 1)	0.937	0.000	80.84	16.12624
SCQ-14	Sport	27	54.111	20.462	1.674 (81, 2)	0.194	0.040	55.593	22.883
	Spiritual	23	63.435	17.386				66.13	17.884
	Control	34	56.618	17.534				53.677	16.64
	Merged yoga	50	58.4	19.491	0.183 (82, 1)	0.670	0.002	60.44	21.204
PA	Sport	27	33.963	6.211	2.351 (81, 2)	0.102	0.055	31.778	7.531
	Spiritual	23	36.87	6.093				33.522	6.57
	Control	34	36.735	4.627				35.176	5.266
	Merged yoga	50	35.3	6.267	1.299 (82, 1)	0.258	0.016	32.58	7.089
NA	Sport	27	19	6.463	0.321 (81, 2)	0.727	0.008	15.852	5.013
	Spiritual	23	20.348	5.365				16.87	4.975
	Control	34	19.353	6.305				18.971	6.956
	Merged yoga	50	19.62	5.962	0.039 (82, 1)	0.844	0.000	16.32	4.971

**MAAS, Mindful Attention and Awareness Scale; BAQ, Body Awareness Questionnaire; SCQ-14, Spiritual Connection Questionnaire; PA, Positive Affect; NA, Negative Affect.**

**FIGURE 1:**
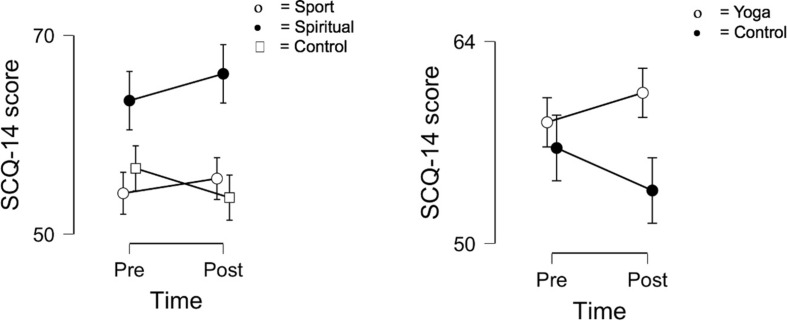
Changes in spirituality in the three groups (**left-hand** side) and in the merged yoga and control group (**right-hand** side). SCQ-14, Spiritual Connection Questionnaire. Error bars indicate 95% confidence intervals.

**FIGURE 2:**
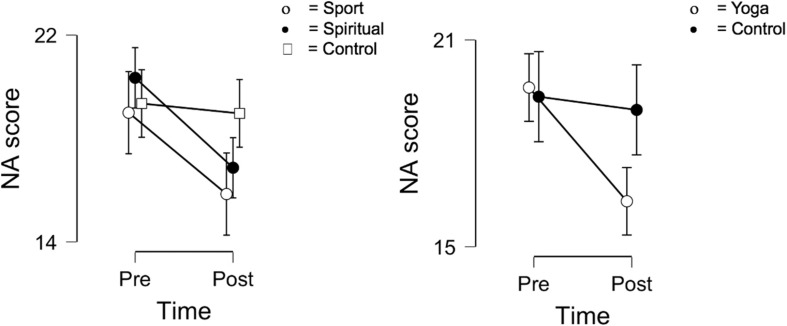
Changes in negative affect in the three groups (**left-hand** side) and in the merged yoga and control group (**right-hand** side). NA, Negative Affect. Error bars indicate 95% confidence intervals.

Results of the 2 × 3 mixed ANOVAs are presented in Table 2. No significant results with respect to mindfulness and body awareness were found. Results of Bayesian analysis were in line with these findings as all BF_10_ indices were less than 1. A significant interaction effect emerged for spirituality [*F*(2, 81) = 3.35; *p* = 0.040; η*_*p*_*^2^ = 0.076], but *post hoc* tests failed to reveal significant differences between groups (Sport-Spiritual: *t* = −1.911; *p_*bonf*_* = 0.179; Sport-Control: *t* = −0.063; *p_*bonf*_* = 1.000; Spiritual-Control: *t* = 1.949; *p_*bonf*_* = 0.164) (Figure 1, left-hand part). Bayesian analysis, however, did not indicate a substantially higher probability for the alternative hypothesis compared to the null hypothesis (*BF*_10_ = 1.054). Concerning positive affect, a significant time effect emerged [*F*(2, 81) = 15.935; *p* < 0.001; η*_*p*_*^2^ = 0.164], which was strongly supported by the Bayesian analysis (*BF*_10_ = 96.677), but no group or interaction effects were present. Significant time effect [*F*(2, 81) = 17.342; *p* < 0.001; η*_*p*_*^2^ = 0.176] and interaction effect [*F*(2, 81) = 3.369; *p* = 0.039; η*_*p*_*^2^ = 0.077] were found for negative affect, but *post hoc* analyses did not show significant between-group differences (Sport-Spiritual: *t* = −0.767; *p_*bonf*_* = 1.000; Sport-Control: *t* = −1.239; *p_*bonf*_* = 0.656; Spiritual-Control: *t* = −0.377; *p_*bonf*_* = 1.000) (Figure 2, left-hand part). Bayesian results strongly supported the former finding (*BF*_10_ = 62.154), however, the latter received only weak support (*BF*_10_ = 1.458).

**TABLE 2:** Results of the 2 × 3 ANOVAs (both frequentist and Bayesian) for the measured variables.

		*F*(2, 81)	*p*	η *_*p*_*^2^	BF_10_
MAAS	Group	1.336	0.269	0.032	0.450
	Time	2.289	0.134	0.027	0.391
	Group * Time	0.497	0.610	0.012	0.146
BAQ	Group	1.276	0.285	0.031	0.346
	Time	0.076	0.784	0.000	0.189
	Group * Time	0.149	0.862	0.004	0.118
SCQ-14	Group	2.377	0.099	0.055	1.045
	Time	0.179	0.673	0.002	0.163
	Group * Time	3.35	0.040*	0.076	1.054
PA	Group	2.573	0.083	0.060	1.008
	Time	15.935	< 0.001***	0.164	96.677
	Group * Time	0.768	0.467	0.019	0.186
NA	Group	0.781	0.461	0.019	0.284
	Time	17.342	< 0.001***	0.176	62.154
	Group * Time	3.369	0.039*	0.077	1.458

MAAS, Mindful Attention and Awareness Scale; BAQ, Body Awareness Questionnaire; SCQ-14, Spiritual Connection Questionnaire; PA, Positive Affect; NA, Negative Affect. *p < 0.05. ***p < 0.001.

Results of the 2 × 2 ANOVAs with the merged yoga group are presented in Table 3. Significant interaction effect emerged for spirituality [*F*(1, 82) = 6.526; *p* = 0.012; η*_*p*_*^2^ = 0.074], weakly supported by the Bayesian analysis (*BF*_10_ = 1.161). The level of spiritual connection increased in the merged yoga group and decreased in the control group after the intervention (Figure 1, right-hand part). Regarding positive affect, a strong time effect was found [*F*(1, 82) = 12.971; *p* < 0.001; η*_*p*_*^2^ = 0.137; *BF*_10_ = 97.488]; the level of positive affect decreased in both groups by the second measurement. There was a significant time effect [*F*(1, 82) = 10.774; *p* = 0.002; η*_*p*_*^2^ = 0.116; *BF*_10_ = 62.294] and an interaction effect [*F*(1, 82) = 6.764; *p* = 0.011; η*_*p*_*^2^ = 0.076; *BF*_10_ = 4.635] for negative affect. The level of negative affect decreased in the yoga group, while it remained basically the same in the control group at the end of the intervention (Figure 2, right-hand part). Concerning mindfulness and body awareness, no significant results were found in either analysis.

**TABLE 3:** Results of the 2 × 2 ANOVAs (both frequentist and Bayesian) for the measured variables.

		*F*(1, 82)	*p*	η *_*p*_*^2^	BF_10_
MAAS	Group	2.032	0.158	0.024	0.447
	Time	1.413	0.238	0.017	0.396
	Group * Time	0.748	0.390	0.009	0.149
BAQ	Group	0.041	0.84	0.001	0.347
	Time	0.008	0.927	0.000	0.168
	Group * Time	0.256	0.614	0.003	0.155
SCQ-14	group	1.067	0.305	0.013	0.849
	Time	0.214	0.645	0.003	0.162
	Group * Time	6.526	0.012*	0.074	1.161
PA	Group	2.788	0.099	0.033	0.996
	Time	12.971	< 0.001***	0.137	97.488
	Group * Time	0.955	0.331	0.012	0.192
NA	Group	0.979	0.325	0.012	0.451
	Time	10.774	0.002**	0.116	62.294
	Group * Time	6.764	0.011*	0.076	4.635

MAAS, Mindful Attention and Awareness Scale; BAQ, Body Awareness Questionnaire; SCQ-14, Spiritual Connection Questionnaire; PA, Positive Affect; NA, Negative Affect. *p < 0.05. **p < 0.01. ***p < 0.001.

Correlations between the change in measured variables within the merged yoga group are presented in Table 4.

**TABLE 4:** Kendall’s tau-b coefficients across the delta variables in the merged yoga group.

*N* = 50	1	2	3	4
1. MAAS	−			
2. BAQ	0.014	−		
3. SCQ-14	0.074	0.283**	−	
4. PA	0.033	–0.013	0.161	−
5. NA	−0.257*	–0.026	–0.037	–0.066

MAAS, Mindful Attention and Awareness Scale; BAQ, Body Awareness Questionnaire; SCQ-14, Spiritual Connection Questionnaire; PA, Positive Affect; NA, Negative Affect. *p < 0.05. **p < 0.01.

Frequentist and Bayesian results consistently indicate a weak to moderate negative correlation between the change in mindfulness and negative affect (*τ_*b*_* = -0.257; *p* = 0.012; *BF*_10_ = 5.462). Another weak to moderate positive association emerged between body awareness and spirituality (*τ_*b*_* = 0.283; *p* = 0.004; *BF*_10_ = 11.174). No other correlation reaching the accepted significance level (*p* < 0.05) or a BF_10_ higher than 1 was found.

## Discussion

The results of the present study indicate that different types of verbal instructions during physically identical beginner level hatha yoga sessions do not result in different outcomes with respect to spirituality, body awareness, mindfulness, and affect among healthy female university participants. However, when data were merged across intervention groups, yoga participants showed a significant increase in spirituality and a decrease in negative affect when compared to the no treatment control group at the end of the 10-session long yoga training. No significant interaction effect for mindfulness, body awareness, and positive affect was found.

To our knowledge, this is the first study that investigates the role of verbal cuing in the overall effect of a yoga intervention. We expected that more holistic (i.e., referring to philosophical and spiritual aspects of yoga practice) verbal information would lead to greater improvements in mindfulness, body awareness, spirituality, and affect than instructions that focused predominantly on the physical aspects of yoga practice. These hypotheses were not supported by the data. Regardless of the instructions provided in the sessions, yoga practice led to favorable outcomes, i.e., an improvement in spirituality and a decrease in negative affect.

These results are partially in accordance with those of a similarly designed intervention study (Smith et al., 2011). At the end of a 7-week-long training, participants in both the “yoga as exercise” and “integrated yoga” groups showed decreased levels of depression and stress and an increased sense of hope, effects that were not found in the control group. Only anxiety decreased exclusively in the integrated yoga group. In the present study, the decrease in negative affect experienced by both intervention groups is in line with these findings (Watson et al., 1988; Gyollai et al., 2011). However, unlike the present study, Smith et al. (2011) found no significant changes in spirituality (i.e., spiritual well-being) in any of the groups. Inconsistencies in the definition, operationalization, and assessment of spirituality across studies can make it hard to compare outcomes in related papers. Studies assessing single aspects of spirituality, such as spiritual readiness, spiritual connection, or conscious interactions/compassion (Dittmann and Freedman, 2009; Büssing et al., 2012a; Csala et al., 2017), reported a favorable relationship between yoga and spirituality. However, Smith et al. (2011) used a more complex scale with two interrelated yet distinct aspects of spirituality (i.e., religious and existential well-being) and found no changes in spirituality following the yoga intervention. To capture changes in more complex measures of spirituality, a longer, more integrative yoga practice may be required.

Yoga practice has special physical and mental characteristics; it integrates sustained muscular activity with inward focus, breath awareness, and synchronization of breath and movement (Impett et al., 2006; Mehling et al., 2011; Govindaraj et al., 2016). These components are expected to result in a mental stillness, self-contemplative state, and reduction of stress and anxiety (Rama et al., 1976; Iyengar, 1991; Collins, 1998; Sengupta, 2012). The decrease in negative affect found among yoga participants in the current study accords with this, since the instructions provided for both intervention groups included components of *Mindfulness, Body awareness*, and *Breathwork* (i.e., focusing on breathing, deep breathing, and linking breathing with movement). Additionally, awareness of breath can facilitate deeper levels of self-awareness (Rama et al., 1976), thus providing a potential explanation for the improvement in spirituality that both of the intervention groups experienced.

A further explanation for our findings may be that yoga can impact psychological functioning “through the body” regardless of verbal cuing. It has been proposed that postural yoga differs from other purely physical activities because it possesses a distinct contemplative or spiritual dimension (Sarbacker, 2014). Sarbacker (2014) proposes that the physical discipline of yoga has mental and spiritual effects regardless of the practitioner’s motivation. In fact, the original purpose of yoga practice was spiritual liberation (Rama et al., 1976; Iyengar, 1991; Baktay, 1992; Sarbacker, 2014). Yogis developed and utilized physical practices like asana, pranayama, or other techniques to embody psychological and spiritual elements through exercise. In yoga, training and transformation of the body is aligned with psychological and spiritual features or changes (Jois, 2002; Sarbacker, 2014). A person’s physical posture is considered a reflection of his or her state of mind; thus, specific poses can also provoke particular feelings and mental content (Rama et al., 1976). Concerning spirituality and negative affect, results of the present study support this view.

An alternative explanation for the comparable change in the two interventional groups pertains to the attitude of the yoga teacher toward the students. Similarly to psychotherapy (client-centered therapy), the authentic, empathetic, and unconditional positive attitude of the instructor offers emotional support and models how to accept and understand oneself and feel connected to others (Rogers, 1951). These “therapeutic” qualities in yoga instructors can be strengthened through the moral principles of yoga (e.g., non-violence, truthfulness, patience, compassion, sincerity) (Iyengar, 1991; Deborah, 2009).

In contrast to our results, a previous study (Delaney and Anthis, 2010) reported that participants in yoga classes that emphasized the “mind” components of practice (e.g., meditation, chanting, yogic principles, and understanding the non-physical self) reported higher levels of body awareness and satisfaction than those in classes with greater “body” focus. However, that study investigated advanced practitioners (average yoga practice was 3.93 h/week for an average of 4.53 years in their most-frequented class) through questionnaires and did not include any other variables measured in the present study. It is possible that differences in verbal instructions provided during physically identical yoga classes were not enough to generate measurable differences between groups due to the effects of novelty in any beginner level course. However, different verbal cues might lead to significant group-level differences during later phases of yoga practice, at an intermediate or advanced level, once the novelty of yoga practice has diminished.

We did not find significant changes in mindfulness and body awareness. Intervention studies (Curtis et al., 2011; Mathad et al., 2017) that used more intense yoga programs than the present study reported significant improvement in mindfulness. Regarding body awareness, studies reporting favorable outcomes (Daubenmier, 2005; Impett et al., 2006; Tihanyi et al., 2016b) investigated participants with previous yoga experience (i.e., on average 6 years and 2 months; 3 years 6 months), more frequent yoga practice (i.e., 4.96 h/week; 2.4 times/week), or more intense intervention (4.4 h/week for 2 months). As mindfulness and body awareness are positively associated with the weekly frequency of yoga practice among advanced students (Tihanyi et al., 2016a), we can assume that once-per-week commitment, the short duration, and the beginner level of the present intervention were not sufficient to evoke such changes. Differential effects among novice and experienced yoga practitioners may also explain why Delaney and Anthis (2010) found group-level differences in body awareness depending on the focus (mind vs. body) of yoga classes among experienced yoga practitioners, while no group-level differences emerged among inexperienced students in the present study.

The correlation analysis of changes in the psychological variables within the merged yoga group showed promising results. The moderate negative correlation between mindfulness and negative affect can be explained in different ways. Mindful attention can positively impact affect (Hölzel et al., 2011; Tihanyi et al., 2016a), however, high levels of negative affect can also diminish mindfulness. In line with the latter case, a decrease in negative affect through yoga practice facilitates mindfulness. As Büssing et al. (2012a) reported that mindfulness, mood, and spirituality are strongly correlated, effects in both directions simultaneously are also feasible. The moderate positive correlation between body awareness and spirituality in the present study supports Dittmann and Freedman’s (2009) finding that spiritual readiness correlates with body awareness, body responsiveness, and body satisfaction. We can assume that a growing focus on the body leads to different levels of self-experience and activates the innate endeavor of wholeness (Mehling et al., 2011); hence, it fosters spirituality. Nevertheless, it is also possible that spirituality drives the relationship between these variables. Exploration of the associations among these constructs, all of which underlie healthy mental functioning, could result in more appropriate design for mental health interventions (Mehling et al., 2011; Büssing et al., 2012a, b; Hendriks et al., 2017).

Regarding positive affect, a significant time effect occurred in that levels of positive affect decreased in all groups toward the end of the intervention. We interpret this result as being due to the university’s academic calendar. At the end of the semester, students have more requirements to complete; thus, levels of positive affect are known to decrease (Greene and Maggs, 2017). However, there was no significant interaction effect between the yoga and control group. As several previous studies (West et al., 2004; Vadiraja et al., 2009; Meissner et al., 2016; Tihanyi et al., 2016b; Csala et al., 2017) denoted a beneficial impact of yoga practice on positive affect, we can suppose that academic responsibilities obscured any favorable effects of yoga practice on positive affect. On the other hand, we can also assume that since yoga practice endeavors to quiet the mind (Iyengar, 1991; Baktay, 1992), it may lead to a decrease in negative affect (i.e., being calm, placid, relaxed), without having a notable influence on positive affect (i.e., making one enthusiastic, alert, excited).

It can be concluded that even a brief beginner level yoga practice on a weekly basis results in significant positive changes in spirituality and negative affect regardless of verbal instruction, but does not lead to improvements in mindfulness, body awareness, and positive affect. However, for a more detailed understanding of the possible effects of different verbal instructions and different components of yoga practice, further empirical studies are needed (Delaney and Anthis, 2010; Park et al., 2018). For future studies, we recommend the inclusion of more intense or longer-term practices to reveal the impact of duration, level, or intensity of exercise, as well as investigating other specific aspects of verbal information during practice. Further exploration of the relationship between the above-mentioned variables, especially spirituality, is also proposed. Generally speaking, spirituality is an under-researched area in psychology, although many authors argue that it is an essential part of healthy functioning as the term “biopsychosocial–spiritual model” suggests (Hatala, 2013; MacDonald, 2013; Taylor et al., 2013; Elias et al., 2015). A better understanding of the distinct effects of specific components of yoga practice can increase the likelihood of developing adequate intervention programs for various health aims.

### Limitations

The present study has limitations with respect to the specific differences between two scripts for giving the verbal instructions. This study focused primarily on the effects of receiving philosophical and spiritual cues vs. more body-related information. However, the two groups had important commonalities, such as body and breath awareness, as well as meeting regularly with the same group and the same instructor. Personal characteristics of the teacher and the interaction between the students and the teacher might have influenced the findings. Moreover, as participants were aware of participating in a study investigating yoga, experimenter bias can also influence the results. Additionally, we did not intend to investigate the effects of common accessories to yoga practice, such as candles, incense, and music; thus, no accessories were used during the yoga sessions. We must mention the relatively large dropout rate and the lack of randomization between the control and intervention groups. However, the two yoga groups were randomized, and strict inclusion criteria and controlling of potential confounding variables (e.g., previous physical activity level; practicing at home was not allowed) were applied to improve the methodology. Students were asked to avoid home practice, though we did not control for this variable.

Furthermore, it is important to note that the control group received no intervention, i.e., the participants of this group did not attend regular weekly meetings. This might have an impact on the differences between the yoga and the control group. However, our main focus was not to measure the effectiveness of yoga practice but to explore the potential effect of verbal cuing during yoga practice.

Finally, the sample size might have limited the results of the study. *Post hoc* comparisons of the significant 2 × 3 ANOVAs did not reveal any significant group differences, however, with more participants, these comparisons may have reached significance.

## Conclusion

More holistic and spiritual verbal instructions during the physically identical yoga sessions did not result in different outcomes with respect to spirituality, body awareness, mindfulness, and affect. Regardless of verbal cues, however, even a brief beginner level traditional hatha yoga intervention led to a significant increase in spirituality and a decrease in negative affect. Practicing yoga might influence psychological functioning through its physical components, at least among novice practitioners. It seems to have beneficial effects even when only the physical elements of yoga practice are emphasized in class.

## Data Availability Statement

The datasets generated for this study are available on request to the corresponding author.

## Ethics Statement

The studies involving human participants were reviewed and approved by the Research Ethics Committee of the Faculty of Education and Psychology at ELTE Eötvös Loránd University. The patients/participants provided their written informed consent to participate in this study.

## Author Contributions

BC, EF, and BT contributed to the assessment of data, while BC and FK processed the data and performed the statistical analyses. BC wrote the first draft of the manuscript. FK wrote sections of the manuscript. RD was responsible for language editing. All authors contributed to read and commented on the last version of the manuscript. All authors contributed to the conception and design of the study.

## Conflict of Interest

The authors declare that the research was conducted in the absence of any commercial or financial relationships that could be construed as a potential conflict of interest.

## Footnotes

1 In yoga, the body is assumed to have five different layers. The first is the physical body, and the second represents the claimed energetic representation of the body (energetic body). The so-called chakras are centers regulating the flow of energy (i.e., prana) throughout the body. A disturbance in the flow of energy is often referred to as an energetic blockage. Yoga practice aims to improve the energy flow in the body so that it circulates without obstruction, resulting in better mental and physical health (Satyananda Saraswati, 2002).

## Appendix

**TABLE 5:** Yoga class plan of the 10 yoga sessions in general (1.5 h each).

Group/Type of asanas	Specific asana(s)
Crossed leg seated position	Sukhasana – inward focus, breath awareness *(always)*
Warming up exercises (Pawanamuktasana series)	movements of the joints: neck, wrist, elbow, shoulder, hip, knee, ankle – e.g., Baddha Konasana *(5–7 short exercises)*
Marjari asana	cat-cow pose and its variations *(always)*
Sunsalutation	12 steps:
	1. Pranamasana
	2. Urdva Hastasana
	3. Uttanasana
	4. Ashwa Sanchalanasana
	5. Kumbhakasana
	6. Asthanga Namaskara
	7. Bhujangasana
	8. Parvatasana
	9. Ashwa Sanchalanasana
	10. Uttanasana
	11. Urdva Hastasana
	12. Pranamasana
	Hands and arms down, repetition with the other side.
	*(1 round from class 3; 2 rounds from class 6)*
Urdhva Mukha Svanasana, Adho Mukha Svanasana	*(from class 2)*
Standing poses	Tadasana, Uttkatasana, Uttanasana, Virabhadrasana 1., Parsvottanasana, Virabhadrasana 2., Parsvakonasana, Trikonasana, Parivritta Trikonasana, Prasaritta Padottanasana *(3–6 of these poses)*
Balancing postures	Vriksasana, Garudasana, Hasta Padangustanasa beginner version, Ardha Chandrasana, Vhirabadrasana 3., Natarajasana *(1–2 of these poses)*
Inversions *(only occasionally)*	Shasnakasna *(class 4)*, Ardha Sirsasana *(classes 7, 8)* Sirsasana *(classes 9, 10)*
Prones poses	Ardha Shalabasana, Shalabasana variations, Bhujangasana, Saral Bhujangasana, Kapotasana, Dhanurasana *(1–2 of these poses)*
Forward bends and spine stretches	Balasana, Paschimottanasana, Janu Sirsasana, Upavista Konasana, Arda Matsyendrasana, Gomukasana *(1–2 of these poses)*
Supine poses	abdominal poses: Navasana and variations, Purvottanasana, Setu Bhandasana, Ustrsasana, Pawanamuktasana, Supta Hasta Pandagustasana, Chakrasana *(1–2 of these poses)*
Inversions	Sarvangasana, Halasana, Matsyasana *(1–3 of these poses)*
Ending pose	Spinal twist and/or Ananda Balasana *(always)*
Savasana	Relaxation for 8–10 min *(always)*

**Yoga classes always followed the same sequence regarding the groups of asanas, however, sessions varied across which specific asana was/asanas were included from the particular group. Sessions were always completely the same for the two different yoga groups.**
